# Persistence of *Stenotrophomonas maltophilia* in Patients with Bacteremia: Incidence, Clinical and Microbiologic Characters, and Outcomes

**DOI:** 10.3390/microorganisms12122477

**Published:** 2024-12-02

**Authors:** Sarah Kim, Sehyeon Ji, David Cho, Ahrang Lee, Hae Seong Jeong, Minji Kim, Seong Eun Kim, Kyung-Hwa Park, Sook In Jung, Uh Jin Kim, Sung Un Shin, Seung Ji Kang

**Affiliations:** 1 Department of Infectious Disease, Chonnam National University Hospital, Gwangju 61469, Republic of Korea; sarahtoya@naver.com (S.K.); david9533@naver.com (D.C.); lar1313@naver.com (A.L.); tndmsemd21@naver.com (H.S.J.); favorofgod@hanmail.net (S.E.K.); iammedkid@naver.com (K.-H.P.); sijung@chonnam.ac.kr (S.I.J.); 2 Department of Infectious Disease, Chonnam National University Hwasun Hospital, Hwasun 58128, Republic of Korea; tpgus37@naver.com (S.J.); ming2md@naver.com (M.K.); astralio@naver.com (U.J.K.); 3 Department of Infectious Disease, Chonnam National University Medical School, Gwangju 61469, Republic of Korea

**Keywords:** biofilm, mortality, persistent bacteremia, *Stenotrophomonas maltophilia*

## Abstract

The risk factors and outcomes associated with persistent *Stenotrophomonas maltophilia* bacteremia are not well-defined. This retrospective cohort study analyzed 214 cases of *S. maltophilia* bacteremia diagnosed between 2005 and 2022 at two university hospitals, focusing on the clinical and microbiologic characteristics and outcomes of persistent bacteremia. Persistent *S. maltophilia* bacteremia, defined as the detection of *S. maltophilia* for ≥5 days after the initial blood culture, occurred in 25.7% of cases and was significantly associated with mechanical ventilation, polymicrobial infections, and increased 30-day mortality. The early administration of appropriate antibiotics reduced the likelihood of persistence. Isolates from persistent cases demonstrated increased biofilm formation. Molecular typing revealed no specific genotype linked to bacteremia persistence. Resistance to trimethoprim-sulfamethoxazole significantly increased over the study period. Our study offers new insights into the under-explored issue of persistent *S. maltophilia* bacteremia, a condition that constitutes a notable proportion of bloodstream infections and carries high mortality.

## 1. Introduction

*Stenotrophomonas maltophilia*, a Gram-negative, non-fermentative bacillus ubiquitously found in the environment, is a significant opportunistic pathogen, particularly in debilitated or immunocompromised patients [1]. *S. maltophilia* infections are associated with high mortality and previous studies report that mortality from bloodstream *S. maltophilia* infection can reach up to 65% [2,3]. Recently, *S. maltophilia* emerged as the third most common non-fermentative Gram-negative bacterium responsible for bacteremia, following *Pseudomonas aeruginosa* and *Acinetobacter* species [4]. A study conducted from 2008 to 2017 reported a 162% increase in the incidence of *S. maltophilia* isolates from patients with lower respiratory tract infection, highlighting the growing clinical relevance of this pathogen [5]. Along with the increasing number of immunocompromised/debilitated patients receiving advanced medical interventions, the increasing use of antibiotics may be contributing to a surge in infections due to *S. maltophilia*, which has a significant level of inherent resistance to various antibiotics.

In patients with cystic fibrosis (CF), *S. maltophilia* is particularly concerning due to its association with recurrent infections and persistent airway colonization, which significantly compromise clinical outcomes [6]. However, the persistent isolation of *S. maltophilia* from the bloodstream has primarily been reported in case studies. While some cases have been associated with endovascular infections known to cause persistent bacteremia, the underlying cause in many cases remains unclear [7,8,9]. Recent studies have shown persistent isolation of *S. maltophilia* from follow-up blood cultures in 20–52% of cases within 2–7 days after the initial positive culture, an unexpectedly high rate for Gram-negative bloodstream infections (GN-BSIs), which are generally considered transient [10,11,12]. This finding suggests that *S. maltophilia* may predispose to persistent bacteremia. However, due to the limitations of previous studies—including small sample sizes (62 and 21 cases, respectively), inconsistent timing of blood culture follow-up, and insufficient clinical data—our understanding of persistent *S. maltophilia* bacteremia remains very limited [10,11].

Considering the high mortality associated with *S. maltophilia* bacteremia and the challenges in managing this infection [13], a deepened understanding of the clinical features and outcomes related to *S. maltophilia* persistence in the bloodstream is essential. To address these gaps, in this study, we aimed to (i) determine the incidence of persistent *S. maltophilia* bacteremia in a relatively large cohort, (ii) identify clinical variables associated with persistent bacteremia, (iii) evaluate the impact of persistent bacteremia on mortality, and (iv) assess whether specific sequence types (STs) or biofilm-forming capacity are associated with persistent bacteremia.

## 2. Materials and Methods

### 2.1. Patient Population and Data Collection

This retrospective cohort study included patients aged ≥ 18 years with *S. maltophilia* bacteremia diagnosed at Chonnam National University Hospital or Chonnam National University Hwasun Hospital from January 2005 to December 2022. Only patients with their first episode of *S. maltophilia* bacteremia were included. Demographic and clinical data were extracted from electronic medical records, including age, sex, existing comorbidities, predisposing medical conditions, the presence of intravascular devices, prior exposure to antibiotics, treatment agents, and outcomes. The Institutional Review Boards of both study hospitals reviewed and approved the study protocol (CNUHH-2020-060, CNUH-2020-095). Informed consent was waived due to the retrospective, observational nature of the study.

### 2.2. Definitions

Neutropenia was defined as an absolute neutrophil count <1000/μL mm^3^ within one week before the onset of bacteremia [14]. Eradicable foci were defined as removable or drainable foci. Polymicrobial infection was determined when an additional microorganism was identified within 24 h before or 24 h after the initial positive culture of *S. maltophilia*. Appropriate antibiotic treatment was defined as the administration of at least one antibiotic to which the isolated *S. maltophilia* strain was susceptible. Healthcare-associated infection was defined as an infection identified >48 h after hospital admission or in the presence of healthcare-associated risk factors such as receiving home and/or ambulatory intravenous therapy, chemotherapy, hemodialysis, wound care, specialized nursing care, or patients who had attended a hospital clinic within the last 30 days; hospitalization in an acute care hospital for ≥2 days within the last 90 days; or residence in a nursing home or long-term care facility [15]. Thirty-day mortality was defined as the percentage of patients who died from any cause within 30 days from the initial isolation of *S. maltophilia* from the blood. Duration of bacteremia was defined as the number of days between the first and last positive blood culture for *S. maltophilia*. Persistent *S. maltophilia* bacteremia was defined as the presence of positive blood cultures for ≥5 consecutive days from the time of the initial positive culture [10,11]. In the comparison of persistent with non-persistent bacteremia, only patients with follow-up blood culture results and who survived >5 days from the date of the first positive blood culture were included. Recurrence was defined as the re-isolation of *S. maltophilia* from the bloodstream after the completion of antibiotic treatment for the initial bacteremia.

### 2.3. Bacterial Identification and Antibiotic Susceptibility Testing Using Automated Systems

Blood cultures were performed using the BACTEC FA system (Becton Dickinson; Sparks, MD, USA) or the BacT/ALERT VIRTUO system (bioMérieux; Hazelwood, MO, USA). For bacterial identification and antibiotic susceptibility testing for cultured organisms, the VITEK system (bioMérieux) or the MicroScan WalkAway (Beckman Coulter; Sacramento, CA, USA) was used. Additionally, bacterial identification was confirmed using matrix-assisted laser desorption/ionization-time of flight mass spectrometry (MALDI-TOF-MS) (Microflex MALDI Biotyper, Bruker Daltonics, MA, USA or ASTA MicroIDSys, ASTA, Suwon, Republic of Korea). All isolates were tested for susceptibility to trimethoprim/sulfamethoxazole (TMP/SMX), while susceptibility to levofloxacin, ceftazidime, and minocycline was tested for a subset of isolates.

### 2.4. Organism Collection and Manual Antibiotic Susceptibility Testing

Of the total 214 cases of *S. maltophilia* bacteremia, the number of stored frozen (−80 °C) and available isolates for further microbiological testing was 139. For these 139 isolates, additional antimicrobial susceptibility testing and biofilm assays were performed. Before testing, frozen isolates were subcultured on blood agar at 37 °C and reverified using MALDI-TOF-MS. Broth microdilution testing was performed for ceftazidime, levofloxacin, TMP/SMX, minocycline, cefiderocol, tigecycline, colistin, and ceftolozane/tazobactam. The susceptibility results were interpreted using CLSI interpretive criteria [16]. For antibiotics with no minimal inhibitory concentration (MIC) interpretation available, the following MIC values were considered to indicate susceptibility based on previous studies: ≤4 mg/L for ceftolozane/tazobactam, ≤2 mg/L for colistin, and ≤2 mg/L for tigecycline [17,18]

### 2.5. Crystal Violet Staining Biofilm Assay

All available isolates (42 in the bacteremia ≥5 days group and 31 in the bacteremia <5 days group) underwent biofilm assays. Biofilm formation was quantitatively assessed using 96-well flat-bottomed polystyrene microplates with minor modification [19,20]. Aliquots of bacterial suspensions (200 μL, 107 CFU/mL) in trypticase soy broth were added to the microplate wells. After incubation at 37 °C for 24 h, the contents of each well were aspirated and washed twice with 200 μL of phosphate-buffered saline (0.01 M, pH 7.2). The remaining attached bacteria were fixed at 60 °C for 1 h and stained with 0.5% crystal violet. The bound dye was then solubilized with 33% glacial acetic acid and the optical density at 570 nm (OD570) was measured using an Epoch Microplate Spectrophotometer (Bio Tek Instruments, Inc.; Winooski, VT, USA). The *Staphylococcus epidermidis*: American Type Culture Collection (ATCC) 35,948 and ATCC 12,228 were used as positive and negative controls, respectively. Means of triplicate OD570 were used for analysis. A cut-off value (ODc) was established as three standard deviations (SD) above the mean OD of the negative control: ODc = average OD of negative control + (3 × SD of negative control). Grouping of the isolates was carried out according to the following criteria: non-biofilm producer (OD < ODc), weak biofilm producer (ODc < OD < 2 × ODc), moderate biofilm producer (2 × ODc < OD < 4 × ODc), and strong biofilm producer (4 × ODc < OD) [21].

### 2.6. Molecular Typing of Cultured Isolates

To determine the genetic identity of several isolates within cases of persistent or recurrent bacteremia, multi-locus sequence typing (MLST) was performed, as described by Kaiser et al. [22]. Briefly, bacterial DNA was first extracted using Cell SV mini kit (GeneAll Biotechnology Co., Ltd., Seoul, Republic of Korea) according to the manufacturer’s recommendations. Polymerase chain reaction (PCR) was carried out for seven housekeeping genes: atpD, guaA, gapA, nuoD, ppsA, mutM, and recA. Bidirectional sequencing was performed using an ABI Prism 3730xl genetic analyzer (Applied Biosystems, Waltham, MA, USA). The obtained sequences were aligned and interpreted according to the PubMLST website recommendations (https://pubmlst.org/, accessed on 15 September 2024). New alleles of the genes were assigned based on the MLST database and the specific ST was determined (http://pubmlst.org/smaltophilia/, accessed on 15 September 2024). When uploading new alleles, the following parameters were used: technology, Illumina, Inc., San Diego, CA, USA; read length, 200–299 base pairs; coverage, 20–49×; and assembly, de novo.

### 2.7. Statistical Analyses

Statistical analyses were conducted using SPSS software, version 27.0 (IBM Corp.; Armonk, NY, USA). Continuous variables were described using medians and interquartile ranges (IQRs). Groups were compared using the Mann–Whitney U test. Categoric variables were analyzed using Fisher’s exact test or the Chi-squared test, depending on the appropriateness of data distribution and sample size. Multivariate analysis was employed, using the Cox proportional hazards regression model, to identify factors associated with mortality or the persistence of bacteremia. All significance tests were two-tailed, with a *p* value ≤ 0.05 indicating statistical significance.

## 3. Results

### 3.1. Clinical Characteristics and Outcomes of S. maltophilia Bacteremia Diagnosed Between 2005 and 2022

A total of 214 cases of *S. maltophilia* bacteremia were identified, most of which (99.6%) were healthcare-associated. Table 1 presents patients’ demographic and clinical characteristics, along with a comparison between 30-day survivors and non-survivors. The median (IQR) patient age was 66.0 (56.0–75.3) years and 61.7% of patients were male. The predominant underlying diseases were solid tumors (31.8%), diabetes (29.9%), and hematologic malignancies (27.6%). Before the onset of *S. maltophilia* bacteremia, a substantial proportion of patients had received antibiotics (89.7%), corticosteroids (44.4%), and chemotherapy (34.6%). At bacteremia diagnosis, 31.3% of patients were undergoing mechanical ventilation and 13.1% of patients were receiving continuous renal replacement therapy (CRRT). Appropriate antibiotics were administered within 48 h to only 13.6% of patients. The 30-day and 90-day mortality rates were 36.9% and 43.5%, respectively.

In the univariate analysis, factors associated with 30-day mortality were hematologic malignancy (hazard ratio [HR] 3.00, 95% confidence interval [CI] 1.62–5.58, *p* < 0.01), corticosteroid therapy within 30 days of bacteremia onset (HR 3.16, 95% CI 1.77–5.63, *p* < 0.01), mechanical ventilation use (HR 1.95, 95% CI 1.08–3.52, *p* = 0.03), CRRT before bacteremia onset (HR 5.01, 95% CI 1.98–12.72, *p* < 0.01), and pneumonia, including empyema (HR 2.34, 95% CI 1.26–4.33, *p* = 0.01). Conversely, the presence of a solid tumor was associated with lower mortality (HR 0.36, 95% CI 0.19–0.70, *p* < 0.01). Moreover, the effective control of infectious foci in patients with eradicable infection sources significantly reduced mortality (HR 0.13, 95% CI 0.05–0.36, *p* < 0.01).

In the multivariate analysis, previous corticosteroid therapy (HR 3.09, 95% CI 1.46–6.51, *p* < 0.01) and CRRT before bacteremia onset (HR 5.37, 95% CI 1.87–15.40, *p* < 0.01) were independently associated with 30-day mortality (Table 2).

### 3.2. Incidence, Clinical Predictors, and Outcomes of Persistent S. maltophilia Bacteremia

The median (IQR) duration of bacteremia was 1 (0–6) days. Bacteremia persisted for ≥3 days in 82 patients (38.3%), ≥5 days in 55 patients (25.7%), and ≥7 days in 41 patients (19.2%). The recurrence of bacteremia occurred in 11 patients (5.1%) following completion of a course of appropriate antibiotics. The median (range) time to recurrence after antibiotic treatment was 16 (8–145) days.

To investigate the characteristics of prolonged bacteremia, we compared patients with bacteremia lasting ≥5 days to patients with bacteremia lasting <5 days (Table 3). Univariate analysis revealed that patients with ≥5 days of bacteremia were more likely to have a central venous catheter (HR 2.63, 95% CI 1.13–6.11, p = 0.03), mechanical ventilation (HR 9.90, 95% CI 3.63–27.02, *p* < 0.01), and CRRT prior to bacteremia onset (HR 4.89, 95% CI 1.00–23.83, *p* = 0.05) and were more likely to have polymicrobial bacteremia (HR 2.40, 95% CI 0.99–5.80, *p* = 0.05). In contrast, patients with cholangitis as the primary infection (HR 0.29, 95% CI 0.09–0.99, *p* = 0.05) and patients who received appropriate antibiotics within 48 h (HR 0.26, 95% CI 0.78–0.87, *p* = 0.03) were less likely to have ≥5 days of bacteremia. Additionally, the 30-day mortality rate was significantly higher in patients with ≥5 days of bacteremia (HR 5.11, 95% CI 1.87–14.00, *p* < 0.01).

In the multivariate analysis, mechanical ventilation prior to bacteremia onset (HR 12.31, 95% CI 3.45–43.94, *p* < 0.01) and polymicrobial bacteremia (HR 3.50, 95% CI 1.12–10.95, *p* = 0.03) were independently associated with ≥5 days of *S. maltophilia* bacteremia. Conversely, the administration of appropriate antibiotics within 48 h was a negative predictor for ≥5 days of *S. maltophilia* bacteremia (HR 0.16, 95% CI 0.03–0.87, *p* = 0.03).

### 3.3. Biofilm-Forming Ability and ST Analysis of S. maltophilia Isolates in Persistent or Recurrent Bacteremia

Biofilm formation assays demonstrated that isolates from the ≥5 days of bacteremia group (*n* = 42) exhibited significantly greater adherence to hydrophobic surfaces in 96-well plates, with a mean optical density of 0.71 ± 0.23, compared to 0.55 ± 0.25 in the <5 days of bacteremia group (*n* = 31) (*p* < 0.01) (Table 4). When categorizing biofilm formation based on the cut-off value (ODc), nearly all isolates in the ≥5 days group were biofilm producers, with only one isolate being a non-producer. Additionally, 42.9% of isolates in this group were moderate biofilm producers. In contrast, 16.1% of isolates in the <5 days group were non-producers and the majority of biofilm-forming isolates in this group were categorized as weak biofilm producers.

MLST was performed for 12 of the 55 cases in the ≥5 days of bacteremia group and for 5 of the 11 recurrent cases, which were selected based on the availability of multiple isolates cultured at different time points within the same case. In 17 cases, seventeen STs were identified, including eight previously reported STs and nine first-identified STs in this study (i.e., ST487-495) (Supplementary Table S1). For persistent bacteremia, all isolates obtained at different time points were identified as the same ST within a case, except in one case where the isolate obtained on day 16 of bacteremia differed in ST from the isolate recovered at the beginning of the bacteremia. In recurrent bacteremia, the STs of the strains isolated from the initial and recurrent episodes were identical in three cases, but different in one case.

### 3.4. In Vitro Antimicrobial Susceptibility

Antimicrobial susceptibility was evaluated for 139 stored isolates using the broth microdilution method (Table 5). Colistin exhibited the highest resistance rate (83.5% of isolates), followed by ceftazidime (72.7%), TMP-SMX (28.1%), and levofloxacin (22.3%). Resistance rates for tigecycline (15.1%), cefiderocol (5.0%), and minocycline (2.2%) were relatively low. Resistance to both TMP-SMX and levofloxacin was found in 10.1% of the isolates. Among the isolates resistant to both TMP-SMX and levofloxacin, eight isolates were resistant to tigecycline, three to cefiderocol, and two to minocycline. A temporal analysis of antibiotic resistance revealed a significant increase in TMP-SMX resistance, reaching 69.2%, during 2000–2022 (*p* < 0.01). No significant differences in antibiotic resistance were observed based on mortality or the duration of bacteremia (Supplementary Tables S2 and S3).

## 4. Discussion

A notable finding in our study was that many patients experienced prolonged *S. maltophilia* bacteremia, with 39% of patients having bacteremia for ≥3 days, 30% for ≥5 days, and 24% for ≥7 days. The persistence of pathogens in the bloodstream is a well-recognized characteristic of *Staphylococcus aureus* bacteremia and candidemia. Previous studies have reported persistence rates ranging from 8–39% in *S. aureus* bacteremia [23,24] and 8–15% in candidemia [25,26]. Additionally, several studies provided substantial evidence on the risk factors and adverse outcomes associated with persistent bloodstream infections caused by these pathogens [27,28]. In contrast, few studies have investigated persistent Gram-negative bloodstream infections (GN-BSIs), as GN-BSIs are generally considered transient [12]. However, recent studies reported the re-isolation of *S. maltophilia* from follow-up blood cultures taken 2–7 days after the initial positive culture in 20–52% of cases [10,11]. These rates are unexpectedly high for GNB-BSIs, but consistent with our study findings, and highlight the risk of persistent *S. maltophilia* bacteremia. To date, there have been no adequate studies investigating the clinical significance of persistent *S. maltophilia* bacteremia. To the best of our knowledge, this is the first study to comprehensively investigate the clinical and microbiologic characteristics of prolonged *S. maltophilia* bloodstream infections.

In our investigation, patients previously supported by mechanical ventilation and with polymicrobial infections (i.e., with severe underlying comorbidities and compromised host immune status) were significantly more likely to experience ≥5 days of bacteremia. Studies evaluating risk factors for persistent GN-BSIs generally highlight severe medical comorbidities as predictors, which is consistent with our findings [12].

In our study, only 13.6% of patients received appropriate antibiotics within the first 48 h, a finding consistent with previous studies [29,30]. This is likely due to the multidrug-resistant nature of *S. maltophilia*, which reduces the likelihood that the empiric choice of antibiotics will be appropriate. In our study, appropriate antibiotic use within 48 h was identified as a negative predictive factor for ≥5 days of bacteremia, but did not have a significant impact on 30-day mortality.

The notion of microbial persisters associated with chronic persistent infections was first reported in the 1940s and has since been identified in several bacterial species [31]. Persisters are a subpopulation of drug-susceptible bacteria that survive in a non-growing or slow-growing state, enabling them to tolerate antibiotics and environmental stresses, such as acidic or nutrient-deprived conditions. This bacterial dormant state is known to play a critical role in infections involving biofilm formation and immune evasion, such as urinary tract infections, cystic fibrosis, and infections related to indwelling medical devices [31]. Previously, bacterial dormancy was thought to exhibit a hierarchy, with a deeper dormancy state termed the viable but non-culturable (VBNC) state. In this state, bacteria were considered non-culturable under standard laboratory conditions but potentially resuscitable in enriched or host-mimicking environments [32]. However, recent studies indicate that VBNC cells are characterized as empty, membrane-enclosed vesicles lacking cytosolic content and metabolic activity, representing the non-viable remnants of bacterial cells. This updated understanding redefines bacterial dormancy as being driven exclusively by persister cells capable of reactivation under favorable conditions [33].

Research on the persistence of *S. maltophilia* remains relatively limited compared to other pathogens [34,35]. However, this organism is known to utilize various virulence factors and associated elements—such as biofilm formation, fimbrial structures, small colony variant formation, and the ability to colonize within murine macrophages—to facilitate immune evasion and persistent infection. These traits are especially likely to contribute to chronic infection in the airways of CF patients [36,37,38]. Considering the potential for these factors to similarly promote persistence in bloodstream infections, we compared the biofilm production ability of *S. maltophilia* isolates from persistent and non-persistent bacteremia cases. Our findings indicate that the ability to produce biofilms was more prominent in isolates recovered from patients with ≥5 days of bacteremia, suggesting that this feature may partly explain the phenotypic characteristics of persistent *S. maltophilia* bloodstream infections. However, as seen in *S. aureus* research, persistent bacteremia is determined by a complex range of genetic and phenotypic factors, and further studies are needed to fully elucidate the specific microbiologic features of *S. maltophilia* that contribute to persistent bloodstream infection [39].

The MLST analysis revealed 17 distinct STs among 17 isolates obtained from cases of ≥5 days or recurrent bacteremia, suggesting that no specific genotype is associated with persistent bloodstream infections. This finding aligns with previous studies showing that *S. maltophilia* isolates from various contexts—such as colonization and chronic infection in CF patients, non-CF patients, and environmental isolates—are genetically highly diverse, with relatively low correlations between genotype and phenotype [40,41]. In most persistent bacteremia cases, the same ST was identified across multiple isolates collected at different time points, suggesting that the infection was caused by the same strain throughout. However, in one persistent case, a different ST was found later in the infection, indicating possible strain variation. Similarly, in recurrent bacteremia, the same ST was generally detected in both the initial and recurrent episodes, except for one case where different strains were identified, suggesting that most recurrent infections were caused by the same strain, while one case may have involved a new strain. Although there are no comparable studies examining whether the same ST is sustained over the infection period in persistent or recurrent *S. maltophilia* bacteremia, our findings align with those of studies on chronic infections in CF, where the same strain has been identified over extended periods [41].

*S. maltophilia* is inherently resistant to various antibiotics. Moreover, there have been growing reports of resistance to previously effective antibiotics, which complicates the treatment of *S. maltophilia* infections [1]. In our study, the overall susceptibility to TMP/SMX was 71.9%, with a marked decrease over time. Although TMP/SMX has traditionally been considered a preferred therapeutic option for *S. maltophilia*, susceptibility rates show significant regional variability, ranging from 77% to 96% in various studies [42,43,44]. Studies from Korea have shown a similar trend of relatively low TMP/SMX susceptibility rates compared to other regions, with approximately 87% susceptibility, which aligns with the lower rates observed in our study [45]. In contrast to studies reporting stable TMP/SMX susceptibility rates over time, other studies, including ours, have documented a significant decrease in susceptibility [5,46]. This trend is concerning, given TMP/SMX’s role as a primary treatment option for *S. maltophilia* infections. In light of this concerning trend, ongoing monitoring of TMP/SMX susceptibility is essential, particularly in regions or patient populations where decreasing susceptibility rates are observed.

This study has several imitations. First, as a retrospective study, not all *S. maltophilia* blood isolates were collected during the study period. As such, microbiologic assessments could not be done in all *S. maltophilia* bacteremia cases. Second, since this was not a prospectively controlled study, there were limitations in evaluating the impact of timely antibiotic administration and infection source control on outcomes. Despite these limitations, the study strengths are the relatively large patient cohort and the simultaneous review of both clinical and microbiologic aspects of *S. maltophilia* bloodstream infection.

## 5. Conclusions

Our study offers new insights into the under-explored issue of *S. maltophilia* persistent bacteremia, a condition that constitutes a notable proportion of bloodstream infections and carries high mortality. These findings highlight the importance of early and appropriate antibiotic treatment when *S. maltophilia* is detected in the blood, as well as the need for the active monitoring of bacterial clearance to potentially improve patient outcomes.

## Figures and Tables

**Table 1:** Clinical characteristics and outcomes of *S. maltophilia* bacteremia: A comparative analysis between 30-day survivors and non-survivors.

Characteristics	Total (N = 214)	Non-Survivor (*n* = 79)	Survivor (*n* = 135)	Unadjusted HR (95% CI)	*p*-Value
Age, years, median (IQR)	66.0 (56.0–75.3)	66.0 (51.0–76.0)	66.0 (57.0–75.0)	1.00 (0.98–1.02)	0.75
Male, *n* (%)	132 (61.7)	48 (60.8)	84 (62.2)	0.94 (0.53–1.66)	0.83
Comorbidities, *n* (%)					
Solid tumor	68 (31.8)	15 (19.0)	53 (39.3)	0.36 (0.19–0.70)	<0.01
Diabetes mellitus	64 (29.9)	21 (26.6)	43 (31.9)	0.78 (0.42–1.44)	0.42
Hematologic malignancy	59 (27.6)	33 (41.8)	26 (19.3)	3.00 (1.62–5.58)	<0.01
Hematopoietic stem cell transplantation	13 (6.1)	8 (10.1)	5 (3.7)	2.93 (0.92–9.29)	0.08
Predisposing conditions, *n* (%)					
Neutropenia	42 (19.6)	20 (25.3)	22 (16.3)	1.74 (0.88–3.45)	0.11
Chemotherapy within past 30 days	74 (34.6)	33 (41.8)	41 (30.4)	1.65 (0.92–2.93)	0.09
Corticosteroid within past 30 days	95 (44.4)	49 (62.0)	46 (34.1)	3.16 (1.77–5.63)	<0.01
Previous exposure to any antibiotics	192 (89.7)	73 (92.4)	119 (88.1)	1.64 (0.61–4.37)	0.32
Mechanical ventilation before bacteremia onset	67 (31.3)	32 (40.5)	35 (25.9)	1.95 (1.08–3.52)	0.03
CRRT before bacteremia onset	28 (13.1)	21 (21.5)	7 (5.2)	5.01 (1.98–12.72)	<0.01
Primary source of infection, *n* (%)					
Catheter-related infection	78 (36.4)	25 (31.6)	53 (39.3)	0.72 (0.40–1.29)	0.26
Pneumonia or empyema	58 (27.1)	30 (38.0)	28 (20.7)	2.34 (1.26–4.33)	0.01
Intra-abdominal infection	19 (8.9)	9 (11.4)	10 (7.4)	1.61 (0.62–4.14)	0.32
Skin and soft tissue infection	11 (5.1)	6 (7.6)	5 (3.7)	2.14 (0.63–7.25)	0.34
Cholangitis	31 (14.5)	5 (6.3)	26 (19.3)	0.28 (0.10–0.77)	<0.01
Unknown	15 (7.0)	3 (3.8)	12 (8.9)	0.41 (0.11–1.48)	0.16
Presence of eradicable infection focus, *n* (%)	93 (43.5)	33 (35.5)	60 (64.5)	0.64 (0.36–1.14)	0.13
Infection focus eradication, *n* (%)	49/93 (52.7)	9/33 (27.3)	40/60 (66.7)	0.13 (0.05–0.36)	<0.01
Appropriate antibiotics within 48 h	29 (13.6)	9 (11.4)	20 (14.8)	0.74 (0.32–1.71)	0.48

CI, confidence interval; CRRT, continuous renal replacement therapy; HR, hazard ratio; IQR, interquartile range.

**Table 2:** Factors associated with 30-day mortality in patients with *S. maltophilia* bacteremia; multivariate analysis.

Characteristics	Adjusted HR (95% CI)	*p*-Value
Solid tumor	0.81 (0.31–2.09)	0.38
Hematologic malignancy	2.40 (0.96–6.02)	0.06
Corticosteroid within past 30 days	3.09 (1.46–6.51)	<0.01
Mechanical ventilation before bacteremia onset	2.14 (0.95–4.85)	0.07
CRRT before bacteremia onset	5.37 (1.87–15.40)	<0.01
Primary source of infection: pneumonia or empyema	1.37 (0.67–2.85)	0.38
Primary source of infection: cholangitis	0.68 (0.18–2.58)	0.57
Corticosteroid within past 30 days	3.09 (1.46–6.51)	<0.01
Mechanical ventilation before bacteremia onset	2.14 (0.95–4.85)	0.07

CI, confidence interval; CRRT, continuous renal replacement therapy; HR, hazard ratio.

**Table 3:** Clinical characteristics and outcomes for patients with ≥5 days versus <5 days of *S. maltophilia* bacteremia.

Characteristics	Bacteremia ≥5 Days (*n* = 55)	Bacteremia <5 Days (*n* = 52)	Unadjusted HR (95% CI)	*p*-Value	Adjusted HR (95% CI)	*p*-Value
Age, years, median (IQR)	64.0 (55.0–74.0)	65.0 (57.0–74.0)	1.00 (0.98–1.03)	>0.9	0.99 (0.96–1.03)	0.56
Male, *n* (%)	31 (56.4)	38 (73.1)	0.48 (0.21–1.00)	0.07	0.55 (0.20–1.50)	0.24
Comorbidities, *n* (%)						
Solid tumor	17 (30.9)	21 (40.4)	0.66 (0.30–1.46)	0.31		
Diabetes mellitus	17 (30.9)	17 (32.7)	0.92 (0.41–2.08)	0.84		
Hematologic malignancy	13 (23.6)	15 (28.8)	0.76 (0.32–1.81)	0.54		
Hematopoietic stem cell transplantation	3 (5.5)	4 (7.7)	0.69 (0.15–3.25)	0.64		
Predisposing conditions, *n* (%)						
Neutropenia	9 (16.4)	10 (19.2)	0.82 (0.30–2.22)	0.70		
Chemotherapy within past 30 days	18 (32.7)	25 (48.1)	0.53 (0.24–1.15)	0.11		
Corticosteroid within past 30 days	27 (49.1)	20 (38.5)	1.54 (0.72–3.33)	0.27		
Presence of central venous catheter	43 (78.2)	30 (57.7)	2.63 (1.13–6.11)	0.03	1.93 (0.52–7.20)	0.33
Mechanical ventilation before bacteremia	31 (56.4)	6 (11.5)	9.90 (3.63–27.02)	<0.01	12.31 (3.45–43.94)	<0.01
CRRT before bacteremia onset	9 (16.4)	2 (3.8)	4.89 (1.00–23.83)	0.05	0.90 (0.12–6.86)	0.92
Primary source of infection, *n* (%)						
Catheter-related infection	26 (47.3)	19 (36.5)	1.56 (0.72–3.38)	0.26		
Catheter removal	17/26 (65.4)	11/19 (57.9)	1.37 (0.41–4.64)	0.61		
Catheter removal within 48 h	1/26 (3.8)	4/19 (21.1)	0.15 (0.02–1.47)	0.10		
Pneumonia or empyema	13 (23.6)	12 (23.1)	1.03 (0.42–2.53)	0.95		
Intra-abdominal infection	6 (10.9)	3 (5.8)	2.00 (0.47–8.45)	0.35		
Skin and soft tissue infection	4 (7.3)	1 (1.9)	4.00 (0.43–37.0.)	0.22		
Cholangitis	4 (7.3)	11 (21.2)	0.29 (0.09–0.99)	0.05	0.71 (0.16–3.07)	0.64
Unknown	2 (3.6)	6 (11.5)	0.29 (0.06–1.50)	0.14		
Presence of eradicable infection focus	33 (60.0)	22 (42.3)	2.05 (0.95–4.42)	0.07	0.69 (0.21–2.30)	0.55
Focus control	17/33 (51.5)	13/22 (59.1)	0.74 (0.25–2.19)	0.58		
Polymicrobial bacteremia	20 (36.4)	10 (19.2)	2.40 (0.99–5.80)	0.05	3.50 (1.12–10.95)	0.03
Appropriate antibiotics within 48 h	4 (7.3)	12 (23.1)	0.26 (0.78–0.87)	0.03	0.16 (0.03–0.87)	0.03
30-day mortality	22 (40.0)	6 (11.5)	5.11 (1.87–14.00)	<0.01		

CI, confidence interval; CRRT, continuous renal replacement therapy; HR, hazard ratio; IQR, interquartile range.

**Table 4:** Comparison of biofilm production capacity between isolates form ≥5 days and <5 days of *S. maltophilia* bacteremia.

	Bacteremia ≥ 5 Days *n* = 42 (%)	Bacteremia < 5 Days *n* = 31 (%)	*p*-Value
OD 570, mean ± SD	0.71 ± 0.23	0.55 ± 0.25	0.01
Categorization of biofilm producers			<0.01
Negative	1 (2.4)	5 (16.1)	
Weak	23 (54.8)	22 (71.0)	
Moderate	18 (42.9)	4 (12.9)	

**Table 5:** Antimicrobial resistance of 139 *S. maltophilia* bloodstream isolates tested by broth microdilution.

	Number (%) of Resistant Strains							
	2005–2007 (*n* = 12)	2008–2010 (*n* = 21)	2011–2013 (*n* = 20)	2014–2016 (*n* = 21)	2017–2019 (*n* = 26)	2020–2022 (*n* = 39)	Total (N = 139)	*p*-Value
TMP/SMX	1 (8.3)	0 (0.0)	0 (0.0)	2 (9.5)	9 (34.6)	27 (69.2)	39 (28.1)	<0.01
Levofloxacin	3 (25.0)	2 (9.5)	6 (30.0)	4 (19.0)	6 (23.1)	10 (25.6)	31 (22.3)	0.68
Minocycline	0 (0.0)	0 (0.0)	0 (0.0)	0 (0.0)	0 (0.0)	3 (7.7)	3 (2.2)	0.16
Tigecycline	2 (16.7)	3 (14.3)	6 (30.0)	2 (9.5)	3 (11.5)	5 (12.8)	21 (15.1)	0.49
Cefiderocol	0 (0.0)	0 (0.0)	1 (5.0)	0 (0.0)	3 (11.5)	3 (7.7)	7 (5.0)	0.33
Ceftazidime	10 (83.3)	15 (71.4)	15 (75.0)	14 (66.7)	19 (73.1)	28 (71.8)	101 (72.7)	0.95
Colistin	9 (75.0)	15 (71.4)	15 (75.0)	19 (90.5)	22 (84.6)	36 (92.3)	116 (83.5)	0.23
TMP-SMX + levofloxacin	1 (8.3)	0 (0.0)	0 (0.0)	2 (9.5)	3 (11.5)	9 (23.1)	15 (10.1)	0.05

TMP-SMX, trimethoprim-sulfamethoxazole.

## Data Availability

The original contributions presented in the study are included in the article/Supplementary Material, further inquiries can be directed to the corresponding authors.

## Supplementary Materials

The following supporting information can be downloaded at: https://www.mdpi.com/article/10.3390/microorganisms12122477/s1, Table S1: Multi-locus sequence types of *S. maltophilia* isolated from persistent or recurrent bacteremia; Table S2: Comparative analysis of antimicrobial resistance in 139 *S. maltophilia* bloodstream isolates: 30-day survivors vs. non-survivors; Table S3: Comparative analysis of antimicrobial resistance in *S. maltophilia* bloodstream isolates: Bacteremia duration of ≥5 days vs. <5 days.
