# Supplementary material for: Persistence of Stenotrophomonas maltophilia in Patients with Bacteremia: Incidence, Clinical and Microbiologic Characters, and Outcomes
Source: Microorganisms. 2024 Dec 2;12(12):2477. doi: 10.3390/microorganisms12122477 (PMC11727890; doi:10.3390/microorganisms12122477)
Supplement: Supplementary file 1 [file microorganisms-12-02477-s001.zip › microorganisms-3268572-supplementary.pdf]

Table S1. Multilocus sequence types of *S. maltophilia* isolated from persistent or recurrent bacteremia.

Patients	Years	Type of bacteremia	Total duration of bacteremia or Days to relapse after completion of initial bacteremia treatment	Sequence type†	*atpD*†	*gapA*†	*guaA*†	*mutM*†	*nuoD*†	*ppsA*†	*recA*†
1	2012	Persistent	13	**492**	116	8	**350**	33	**158**	**211**	158
2	2013	Persistent	15	28	4	3	2	5	9	6	9
3	2013	Persistent	15	**487**	2	**178**	76	45	63	69	57
4	2013	Persistent	16	**490**	**149**	**179**	**349**	**181**	3	8	**170**
				**491**	**149**	**179**	**349**	85	3	8	**170**
5	2015	Persistent	21	**489**	100	**177**	**348**	**180**	111	182	**169**
6	2017	Persistent	28	24	9	21	28	26	15	18	3
7	2017	Persistent	14	**495**	**150**	**180**	**354**	33	4	200	158
8	2017	Persistent	12	208	4	76	155	5	70	84	9
9	2017	Persistent	24	115	3	1	84	57	25	82	6
10	2018	Persistent	21	496	94	20	229	25	14	17	3
11	2018	Persistent	18	**493**	5	76	**351**	79	70	17	9
12	2018	Persistent	38	**494**	13	28	**352**	**182**	128	143	22
13	2010	Recurred	12	145	82	92	114	69	66	100	81
14	2013	Recurred	7	77	2	66	76	45	63	69	57
15	2013	Recurred	86	24	9	21	28	26	15	18	3
				**488**	10	27	**353**	23	30	**212**	98
16	2014	Recurred	6	**487**	2	**178**	76	45	63	69	57
17	2017	Recurred	137	94	71	74	90	56	2	3	5

†Bold faces indicates newly identified allele and sequence types

Table S2. Comparative analysis of antimicrobial resistance in 139 *S. maltophilia* bloodstream isolates: 30-day survivors vs. non-survivors.

Antimicrobial agents	Total n=139 (%)	Non-survivors n=47 (%)	Survivors n=92(%)	*P*
TMP/SMX	39 (28.1)	13 (27.7)	26 (28.3)	>0.9
Levofloxacin	31 (22.3)	12 (25.0)	19 (20,7)	0.53
Minocycline	3 (2.2)	0 (0)	3 (3.3)	0.55
Tigecycline	21 (15.1)	11 (23.4)	10 (10.9)	0.78
Cefiderocol	7 (5.0)	1 (2.1)	5 (5.4)	0.66
Ceftazidime	101 (72.7)	32 (68.1)	69 (75.0)	0.42
Colistin	116 (83.5)	40 (85.1)	76 (82.6)	0.23
TMP-SMX & Levofloxacin	15 (10.1)	7 (14.9)	8 (8.7)	0.27

TMP-SMX, trimethoprim-sulfamethoxazole

Table S3. Comparative analysis of antimicrobial resistance in *S. maltophilia* bloodstream isolates: Bacteremia duration of ≥5 days vs. < 5 days

Antimicrobial agents	Total<br>n= 73 (%)	Bacteremia ≥5 days<br>n=42 (%)	Bacteremia < 5 days<br>n=31(%)	*P*
TMP/SMX	20 (27.4)	10 (23.8)	10 (32.3)	0.44
Levofloxacin	18 (24.7)	11 (26.2)	7 (22.6)	0.79
Minocycline	1 (1.4)	0 (0)	1 (3.2)	0.43
Tigecycline	11 (15.1)	8 (19.0)	3 (9.7)	0.34
Cefiderocol	4 (5.5)	1 (2.4)	3 (9.7)	0.31
Ceftazidime	52 (71.2)	27 (64.3)	25 (80.6)	0.19
Colistin	63 (86.3)	35 (83.3)	28 (90.3)	0.50
TMP-SMX & Levofloxacin	11 (15.1)	7 (16.1)	4 (12.9)	0.75

TMP-SMX, trimethoprim-sulfamethoxazole
